# *IL1B* polymorphism is associated with essential tremor in Chinese population

**DOI:** 10.1186/s12883-019-1331-5

**Published:** 2019-05-15

**Authors:** Jie Chen, Pei Huang, Yachao He, Junyi Shen, Juanjuan Du, Shishuang Cui, Shengdi Chen, Jianfang Ma

**Affiliations:** Department of Neurology & Co-innovation Center of Neuroregeneration, Ruijin Hospital Affiliated to Shanghai Jiaotong University School of Medicine, Shanghai, 200025 China

**Keywords:** Essential tremor, Genetic risk factors, SNPs, *IL1B*, Restless legs syndrome

## Abstract

**Background:**

The aim of the study was to investigate the genetic risk factors of essential tremor (ET) in Chinese Population.

**Methods:**

A total of 225 ET patients (25 ET patients also had restless legs syndrome (RLS) and were excluded from final analysis) and 229 controls were recruited. The diagnosis of ET was based on the Consensus Statement of the Movement Disorders Society on tremor. Polymerase chain reaction (PCR) and sequencing were used to detect 12 single nucleotide polymorphisms (SNPs) in seven candidate genes for RLS (*HMOX1, HMOX2, VDR, IL17A, IL1B, NOS1 and ADH1B).*

**Results:**

We found that one SNP was associated with the risk of ET in Chinese population after adjusting for age and gender: rs1143633 of *IL1B* (odds ratio [OR] =2.57, *p* = 0.003, recessive model), and the statistical result remained significant after Bonferroni correction. Then, we performed a query in Genotype-tissue Expression (GTEx), Brain eQTL Almanac (Braineac) databases and Blood expression quantitative trait loci (eQTL) browser. The significant association was only found between genotype at rs1143633 and *IL1B* expression level of putamen and white matter in Braineac database, which was more prominent with homozygous (GG) carriers.

**Conclusions:**

Our study firstly reported the association of *IL1B* polymorphism with the risk of ET in Chinese population. However, the association might only suggest a marker of *IL1B* SNP associated with ET instead of the casual variant. Further studies are needed to confirm our finding.

**Electronic supplementary material:**

The online version of this article (10.1186/s12883-019-1331-5) contains supplementary material, which is available to authorized users.

## Background

ET is a clinical disorder of unknown cause characterized by tremor (postural and/or kinetic) predominantly in both upper limbs and other body parts involved occasionally [1].It is not uncommon to notice an overlap of ET with RLS (characterized by dysaesthesias usually in the calves, associated with an irresistible urge to move these limbs) in some families. Clinical surveys have shown that more than half of the ET or RLS patients report at least one first degree relative was affected with either or both of these diseases [2, 3]. Dopamine transporter (DAT) binding in the striatum was shown to be decreased in some RLS patients [4] or ET patients [5], suggesting a possible dopamine dysregulation in both diseases. Genetic factors play an important role in the pathogenesis of both ET and RLS [6–11].Genome wide association study (GWAS) showed that several single nucleotide polymorphisms (SNPs) were associated with the risk of RLS, including *MEIS1, BTBD9, PTPRD, MAP2K5, TOX3* and Intergenic region of *2p14* [12–16]. Recently, we tested those SNPs in Chinese ET and RLS patients and interestingly found a haplotype of *MAP2K5/SKOR1* was both associated with ET and RLS, suggesting a possible genetic link between RLS and ET [17, 18].

Several studies have investigated other candidate genes for RLS, including *HMOX1, HMOX2, VDR, IL17A, IL1B, NOS1, ADH1B and GABRR3*. Garcia-Martin et al. found *HMOX1* rs2071746 was associated with the risk of RLS in the Spanish population [19]. The fact that *HMOX2* rs4786504C allele contributes to high-altitude adaption in Tibetans by leading to a more efficient breakdown of heme also makes us wonder if *HMOX2* could be a risk factor for Chinese RLS [20]. In addition, *VDR* rs731236 SNP has been found associated with RLS [21]. Previous association of *IL1B* rs1143643, rs1143634 or rs1143633 and *IL17A* rs8193036 has been reported in RLS patients with HIV infection [22]. Jimenez-Jimenez et al. analyzed the possible relationship of two common SNPs (rs6413413 and rs1229984) in *ADH1B* with the risk for RLS or Parkinson’s disease and found that rs1229984 SNP was associated with the risk for not only RLS [23], but also with the risk for Parkinson’s disease in women [24]. Furthermore, two SNPs (rs7977109 and rs693534) in *NOS1* have been found associated with German RLS, and only rs7977109 remained significant after correction of multiple testing, while both SNPs were not related to RLS in Spanish population [25, 26]. A recent report described an association between *GABRR3* and the risk for RLS [27]. Among these possible candidate genes for RLS, *HMOX1* rs2071746 and *HMOX2* rs1051308 polymorphisms have been found associated with ET patients in a Spanish population [28], while lack of association of *GABRR3* [29] or *ADH2* [30] with ET was described in previous studies.

Therefore, in this study, we selected 12 SNPs (*HMOX1* rs2071746, *HMOX2* rs4786504, *HMOX2* rs1051308, *VDR* rs731236, *IL17A* rs8193036, *IL1B* rs1143643, *IL1B* rs1143634, *IL1B* rs1143633, *NOS1* rs693534, *NOS1* rs7977109, *ADH1B* rs6413413 and *ADH1B* rs1229984) within seven suspected RLS risk genetic loci (*HMOX1, HMOX2, VDR, IL17A, IL1B, NOS1 and ADH1B*), which had not been tested in Chinese ET patients yet, to further investigate the relationship of these genetic risk factors with ET in Chinese population.

## Methods

### Study population

Two movement disorder specialists independently examined the suspected ET cases by using the standardized tremor examination described by Louis et al. [31, 32] and a full neurological examination was also performed to exclude Parkinsonism and other movement disorders. When there was disagreement between the two movement disorder specialists, a senior movement disorder specialist would evaluate the individuals and make the final diagnosis. The clinical diagnosis of ET was in accordance with the Consensus Statement of the Movement Disorders Society in 1998 [33]. Participants with parkinsonism, drug induced tremors, cerebellar tremor, dystonia and tremors with hyperthyroidism were all excluded from the study. As it is not uncommon for co-morbidity of RLS with ET, an RLS specialist interviewed all those ET patients, and diagnosis of RLS was made based on revised International RLS Study Group diagnostic criteria [34]. Control subjects were recruited through community population and evaluated by RLS and movement disorder specialists for exclusion of ET and RLS. All participants signed consent forms and this study was approved by the ethic committee of Ruijin Hospital affiliated to Shanghai Jiao Tong University School of Medicine.

### DNA preparations and genotyping

About 3 mL blood samples were collected from ET patients and controls. DNA was extracted using phenol-chloroform-isopropyl alcohol method [35]. PCR and extension primers were designed using Primer5 software. SeqMan software was used to identify rs4786504, rs6413413 and rs1229984. Detail of primers and reaction condition of those 3 SNPs were described (See, Additional file 1: Tables S1-S3). Genotyping of other 9 variants was performed using Multiplex SNaPshot. The PCR products were purified by phosphorylase (FastAP) and exonuclease I (EXO I) and extended with ABI SNaPshot Multiplex kit. The elongated product was purified by phosphorylase (FastAP) and loaded on ABI3730xl. SNP typing was analyzed using GeneMapper 4.0 (Applied Biosystems). Detail of primers and reaction condition of those 9 SNPs were also described (See Additional file 1: Tables S4S-6).

### Statistical analysis

Statistical analysis was performed using SAS (version 9.4 TS1M2; SAS Institute Inc., Cary, NC) software package. Student *t* test was used to compare the differences of age between ET sufferers and non-ET/RLS controls. Chi-Square test was used to compare the differences in the gender proportions and test the Hardy-Weinberg equilibrium (HWE) in the total cohort. Alleles of all SNPs distribution between ET patients and controls were also tested by Chi-square test. Risk analysis was performed by logistic regression and odds ratio (OR) was calculated with 95% confidence intervals (CI) for each SNP according to dominant or recessive models, after adjusting for age and sex. Dominant and recessive models were performed based on the assumption of an autosomal-dominant or autosomal-recessive mode of inheritance with incomplete penetrance. Multiplicative terms between age/sex and each SNP were also included in the logistic regression model. Haploview software was used to test linkage disequilibrium (LD) of SNPs in the same chromosome (Additional file 2). Haplotype analysis was also tested by haploview. R (version 3.3.1) was used to correct *p* values by Bonferroni method. Genetic power was calculated by Power and Sample Size Calculations (version 3.1.2). Expression quantitative loci were examined in three different databases: GTEx [36], Blood eQTL browser [37] and Braineac databases [38].

## Results

Totally, 225 ET patients and 229 normal controls were enrolled in this study. Demographic feature of our samples showed that there are no significant differences of age and gender between ET patients and controls. One hundred of total recruited ET (44.44%) had a positive family history for ET. 25 ET patients (11.11%) were also diagnosed with RLS (Additional file 3).

In the first place, we excluded those 25 ET patients also diagnosed with RLS in order to exclude the effect of RLS comorbidity on the association study of SNPs with ET. No significant difference of age and gender was found between 200 ET patients without RLS and 229 controls (Table 1). Among 12 SNPs, all were in HWE except for two SNPs, rs4786504 of *HMOX2* and rs6413413 of *ADH1B* (all were rs6413413AA genotype). Call rates of those 10 SNPs were all > 95%. After adjusting for age and gender, two SNPs of *IL1B* were shown associated with the risk of ET in our cohort (rs1143643: OR = 1.86, *p* = 0.017, recessive model; rs1143633: OR = 2.57, *p* = 0.003, recessive model). However, only rs1143633 of *IL1B* in a recessive model remained statistical significant after correcting *p* value by Bonferroni method (p_corrected_ = 0.024) (Table 2). The effect of age and sex, and their interaction with the studied SNPs in the logistic regression analysis were shown in Additional file 4. We only found significant interactions of sex with *NOS1* rs693534 and *ADH1B* rs1229984 in the dominant model and interaction between sex and *HMOX2* rs1051308 in the recessive model. In order to further exclude gender confounders, we performed a subgroup analysis and found that the association between rs1143633 of *IL1B* and ET remained significant (Additional file 5). No significant difference of age between ET patients and controls were found among female subgroup and male subgroup (Additional file 6).

**Table 1:** Demographic Information of Cases (without RLS) and Controls

	ET sufferers without RLS (*N* = 200)	Non-ET/RLS controls (*N* = 229)	*P* value
Gender, female, N (%)	100 (50.00)	136 (59.39)	0.051
Age, mean (SD), years	65.83 (7.92)	64.30 (13.30)	0.156
Familial history, N (%)	100 (50)	–	–

*ET* essential tremor. *RLS* restless legs syndrome

**Table 2:** Association of SNPs of candidate genes and odds ratio to ET risk (ET without RLS)

Gene	SNP	HWE *p* value	MAF (case/control)		Allele			
					Minor allele	OR	95%CI	*p*
*HMOX1*	rs2071746	0.701	0.44/0.46		A	0.90	(0.69, 1.18)	0.452
*HMOX2*	rs4786504	0.031	–		–	–	–	–
	rs1051308	0.983	0.37/0.37		G	1.00	(0.75, 1.32)	0.983
*VDR*	rs731236	0.255	0.07/0.05		C	1.47	(0.81, 2.65)	0.203
*IL17A*	rs8193036	0.735	0.30/0.30		T	1.04	(0.77, 1.39)	0.809
*IL1B*	rs1143643	0.591	0.46/0.43		G	1.13	(0.86, 1.48)	0.389
	rs1143634	0.431	0.04/0.02		T	1.64	(0.72, 3.75)	0.232
	rs1143633	0.272	0.38/0.34		G	1.22	(0.92, 1.61)	0.171
*NOS1*	rs693534	0.910	0.27/0.27		A	1.03	(0.76, 1.40)	0.858
	rs7977109	0.828	0.22/0.23		G	0.96	(0.69, 1.32)	0.781
*ADH1B*	rs6413413	*–*	*–*		–	–	–	–
	rs1229984	0.729	0.33/0.32		G	1.07	(0.80, 1.43)	0.644
Gene	SNP	Dominant Model (adjusted)			Recessive Model (adjusted)			Genetic Power
		OR	95%CI	*p*	OR	95%CI	*p*	
*HMOX1*	rs2071746	0.86	(0.56, 1.32)	0.503	0.90	(0.55, 1.47)	0.671	0.084
*HMOX2*	rs4786504	–	–	–	–	–	–	–
	rs1051308	0.93	(0.62, 1.37)	0.701	1.09	(0.61, 1.96)	0.762	0.050
*VDR*	rs731236	1.51	(0.82, 2.80)	0.188	–	–	–	0.394
*IL17A*	rs8193036	0.98	(0.66, 1.44)	0.909	1.22	(0.64, 2.34)	0.549	0.053
*IL1B*	rs1143643	0.87	(0.57, 1.33)	0.516	1.86	(1.12, 3.11)	0.017	0.095
	rs1143634	1.68	(0.71, 3.96)	0.234	–	–	–	0.629
	rs1143633	1.01	(0.68, 1.50)	0.948	2.57	(1.38, 4.81)	0.003	0.127
*NOS1*	rs693534	0.96	(0.65, 1.41)	0.829	1.33	(0.63, 2.82)	0.455	0.051
	rs7977109	0.98	(0.66, 1.46)	0.938	0.74	(0.29, 1.86)	0.519	0.054
*ADH1B*	rs6413413	*–*	*–*	*–*	–	–	–	–
	rs1229984	0.99	(0.67, 1.46)	0.946	1.58	(0.76, 3.25)	0.219	0.063

Furthermore, we determined whether the statistical significance of *IL1B* still remained when ET patients with concomitant RLS were included. Among 12 SNPs, only rs1143633 of *IL1B* in a recessive model remained statistical significant after Bonferroni correction (OR = 2.63, *p* = 0.002), which was consistent with our former result (Additional file 7). The distribution of rs1143633 genotypes and allelic variants in ET patients with or without concomitant RLS and controls are shown in Table 3. The haplotype analysis showed that no haplotype block in *IL1B* and *NOS1* was associated with ET patients (Additional file 8). The effect of age and sex, and their interaction with each SNP in the logistic regression model were similar to when ET patients with concomitant RLS were excluded (Additional file 9). In the further subgroup analysis (female/male cohort), there was no significant difference of age between ET patients and controls among female cohort and male cohort (Additional file 6). The association between rs1143633 of *IL1B* and ET was only shown significant in female cohort (*p* = 0.016) instead of male cohort (*p* = 0.052), after adjustment for age (Additional file 5).

**Table 3:** *ILIB* rs1143633 genotypes and allelic variants of ET patients and controls

rs1143633	All ET patients (*n* = 225)	Controls (*n* = 229)	ET patients without concomitant RLS (*n* = 200)
SNP Call rate	97.3%	99.1%	97.5%
Genotypes			
GG	38 (17.4%)	17 (7.5%)	33 (16.9%)
GA	95 (43.4%)	120 (52.9%)	84 (43.1%)
AA	86 (39.2%)	90 (39.6%)	78 (40.0%)
Alleles			
G	171 (39.0%)	154 (33.9%)	150 (38.5%)
A	267 (61.0%)	300 (66.1%)	240 (61.5%)
Recessive model			
GG	38 (15.1%)	17 (7.5%)	33 (16.9%)
AA+GA	181 (84.9%)	210 (92.5%)	162 (83.1%)

The values in each cell represent: number (percentage)

Last, we performed a query in GTEx, Braineac and Blood eQTL browser to determine any phenotypic interest of rs1143633. Brain eQTL data were collected through GTEx and Braineac databases. No significant association between genotypes at rs1143633 and brain *IL1B* expression level was found in GTEx, while in Braineac database the G allele increased the expression in putamen (*p* = 8.70 × 10^− 3^, with probe set 2,571,522) and white matter (*p* = 8.50 × 10^− 4^, with probe set 2,571,524), and a more prominent increase were seen in homozygous carriers (Additional file 10). In blood, we did not observe a cis-eQTr trans-eQTL effect for this SNP on *IL1B*.

## Discussion

This is the first study demonstrating a possible association of *IL1B* rs1143633 with ET in Chinese population, among 12 SNPs harbored in seven possible RLS genes loci, including *HMOX1, HMOX2, VDR, IL17A, IL1B, NOS1* and *ADH1B*. In further subgroup analysis to exclude gender confounders, the significance still remained in both male and female cohort when only ET patients without RLS were included.

*IL1B* gene encodes for the proinflammatory cytokine interleukin-1β (IL-1β), which belongs to member of IL-1 family and is produced by several cell types including blood monocytes, tissue macrophages and cells of the central nervous system [39]. It has been found mediating chronic inflammation in neurodegenerative conditions, such as Parkinson’s disease [40, 41], Amyotrophic lateral sclerosis [42], Alzheimer’s disease [43, 44] and neurodegenerative process in inflammatory disease such as multiple sclerosis [45].

The possible pathophysiology of IL-1β in ET remains largely unclear. Two main pathogenetic mechanisms of ET have been proposed [46, 47]. The traditional olivary model attributes ET to a pathological pacemaker in the inferior olivary nucleus that drives tremor. Another model is cerebellar degenerative model which suggested that the degeneration of Purkinje neurons and connected neuronal populations played important role in the pathogenesis of ET. Based on cerebellar degenerative model, IL-1β might be involved in ET through an immune-based neurodegenerative pathogenesis. In line with this hypothesis, IL-1β can also cause an imbalance between the GABAergic and glutamatergic synaptic transmission at Purkinje cell synapses in experimental autoimmune encephalomyelitis (EAE) cerebellum, which are early events triggering secondary excitotoxicity and inflammatory neurodegeneration in EAE disease [48, 49]. However, we should be very cautious with this hypothesis as the association of IL-1β with ET in our study might only suggest a marker of *IL1B* SNP, and did not imply any inflammation pathogenesis in ET. Besides, we only found a significant association of genotype at rs1143633 with *IL1B* expression level in putamen and white matter, not cerebellum.

No significant differences were found either in the frequencies of genotypes or in the frequencies of the allelic variants of other SNPs harbored in possible RLS genes loci, including *HMOX1, HMOX2, VDR, IL17A, IL1B, NOS1* and *ADH1B*. Our study did not replicate the previously reported association of*HMOX1* rs2071746 and *HMOX2* rs1051308 polymorphisms with ET, which could be due to different ethnic populations or small sample of our study.

Some limitations of our study should be noticed. Firstly, the number of ET patients and controls recruited in our study is relatively small. Therefore, the power of our study is low (Table 2 and Additional file 7), which could lead to inflation of the effect size and potentially mimic the positive signal. Similar studies with big sample size or GWAS are warranted in the future to confirm our findings in Asians or other populations. Secondly, there could be population sub-stratification that was not corrected for due to using self-reported ethnicity instead of genetic ancestry. Furthermore, 9 variants in our study were genotyped by using Multiplex SNaPshot, which was less reliable compared to Sanger sequencing. Lastly, some ET might have a subclinical dopaminergic deficiency which could introduce an enrollment bias of ET in our cohorts. Although the diagnosis of ET is performed by two experienced movement disorder specialists, it would be better to perform a DAT-SCAN to exclude this possibility. Unfortunately, due to financial concerns, we did not perform DAT-SCAN in our ET patients.

## Conclusion

We found a significant association of *IL1B* rs1143633 polymorphism in the recessive model with the risk for ET in Chinese population. However, the results should be taken with caution because segregation analysis of familial ET often suggests an autosomal dominant inheritance and there was no significant difference in the frequencies of allelic variants in our study. In addition, after a query in GTEx, Braineac and Blood eQTL browser, significant association between genotype at rs1143633 and *IL1B* expression level was only found in Braineac database in putamen and white matter, which was inconsistent with the main hypothesis of ET as a disorder with cerebellum involvement. This significant SNP was a marker and more likely not the casual variant, but in linkage disequilibrium with the casual variant. Furthermore, it is still too early to draw the conclusion that ET has relation with RLS from genetic point of view, since association of *IL1B* has only been reported in RLS patients with HIV infection. More studies are certainly needed in the future to replicate our finding and investigate the true causal variant.

## Additional files

Additional file 1: Detail of primers and reaction condition of 12 selected SNPs. (DOCX 15 kb)

Additional file 2: linkage disequilibrium (LD) of SNPs in the same chromosome. (DOCX 13 kb)

Additional file 3: Demographic Information of Cases (all ET patients) and Controls (DOCX 12 kb)

Additional file 4: The effect of age and sex, and their interaction with each SNP (ET without RLS) (DOCX 19 kb)

Additional file 5: Subgroup analysis of association between rs1143633 and ET risk in recessive model (DOCX 15 kb)

Additional file 6: Comparison of age between ET patients and controls in subgroup analysis (DOCX 13 kb)

Additional file 7: Association of SNPs of candidate genes and odds ratio to ET risk (all ET patients) (DOCX 17 kb)

Additional file 8: Haplotype association study of *IL1B* and *NOS1 (DOCX 13 kb)*

Additional file 9: The effect of age and sex, and their interaction with each SNP (all ET patients) (DOCX 19 kb)

Additional file 10: Effects of rs1143633 genotype on brain *IL1B* expression level. (DOCX 28 kb)

## Abbreviations

Braineac: Brain eQTL Almanac
CI: Confidence intervals
DAT: Dopamine transporter
EAE: Experimental autoimmune encephalomyelitis
eQTL: Expression quantitative trait loci
ET: Essential tremor
GTEx: Genotype-tissue Expression
GWAS: Genome wide association study
HWE: Hardy-Weinberg equilibrium
IL-1β: interleukin-1β
LD: Linkage disequilibrium
OR: Odds ratio
PCR: Polymerase chain reaction
RLS: Restless legs syndrome
SNP: Single nucleotide polymorphism
