# Supplementary material for: IL1B polymorphism is associated with essential tremor in Chinese population
Source: BMC Neurol. 2019 May 15;19:99. doi: 10.1186/s12883-019-1331-5 (PMC6518722; doi:10.1186/s12883-019-1331-5)
Supplement: Supplementary file 1 — Detail of primers and reaction condition of 12 selected SNPs. (DOCX 15 kb) [file 12883_2019_1331_MOESM1_ESM.docx]

Table S1 Primer Sequences of 3 SNPs

| Gene | SNPs | Primer Type | Primer Sequences (5’→3‘’) | Length (bp) |
| --- | --- | --- | --- | --- |
| *HMOX2* | rs4786504 | Forward | CTCAAGGTCCTGAGGGGAATG | 21 |
|  |  | Reverse  SEQ | TTCCCTCTCGTTGCAGCG | 18 |
|  |  |  | CCTGACCAACATGGAGAAAC | 20 |
| *ADH1B* | rs6413413 | Forward | GACTGAATAACCTTGGGGATAAAC | 24 |
|  |  | Reverse  SEQ | TCTCTCATTGCCTTGGTTTCCTTA |  |
|  |  |  | TCTCTCATTGCCTTGGTTTCCTTA |  |
|  | rs1229984 | Forward | CTGAATAACCTTGGGGATAAACTG | 24 |
|  |  | Reverse  SEQ | CTCTCATTGCCTTGGTTTCCTTAT |  |
|  |  |  | GTAGGGATTAGTAGCAAAACCCTC |  |

Table S2 The PCR Reaction System of rs4786504

|  | Volume |  |
| --- | --- | --- |
| ExTaq premix | 10μL |  |
| ddH_2_O | 8μL |  |
| Template | 1μL |  |
| Forward Primer (10 μmol/L) | 0.5μL |  |
| Reverse Primer (10 μmol/L) | 0.5μL |  |

Reaction condition: 94℃3min+ (94℃30s+55℃30S+72℃1min)*35+72℃10min

Table S3 The PCR Reaction System of rs6413413 and rs1229984

|  | Volume |  |
| --- | --- | --- |
| Tiandz PCR mix | 12.5μL |  |
| ddH_2_O | 9.5μL |  |
| Template | 1μL |  |
| Forward Primer (10 p) | 1μL |  |
| Reverse Primer (10 p) | 1μL |  |

Reaction condition: 94℃5min+ (94℃30s+55℃30S+72℃1min)*38+72℃10min

Table S4 Primer Sequences of other 9 SNPs

| SNP | F | R | Extension |
| --- | --- | --- | --- |
| rs1143643 | CTCCTCAGCATTTGGCACTAAGTTT | CCAGAAAGGAAACTGACGAGCAGGT | AGAAGGGCTCTTTTAATAATCACAC |
| rs2071746 | TAAGTTCCTGATGTTGCCCACC | CGTCCCAGAAGGTTCCAGAAAG | GATGTTGCCCACCAGGCT |
| rs8193036 | AGAGTACAGAGAAAAGAACCGCTAACT | GGAATTGAAAATGGGGATAGAGACT | GATAGAGACTGGACAAAGGTGATG |
| rs7977109 | GCTCAGGCTGGTGACGGTAG | GATAATAAGGTTGTTGCTTGGCACAT | AGAACTGCGAAGGACTAATACTGAA |
| rs693534 | CCAGGATATCACATTCTTCAATCAGG | CTGTATTAACCAAACGCAATTCATT | CTCTGGTCAGATTCTATCAAAGACA |
| rs1143634 | CATCAGACTTTGACCGTATATGCTCAG | TGATCGTACAGGTGCATCGT | AGCCTCGTTATCCCATGTGTC |
| rs731236 | CCTTCTTCTCTATCCCCGTGC | TAGAGCAGGTGGCTGCCCG | CAGGACGCCGCGCTGAT |
| rs1051308 | CTTGGGTGCGGTTTGCTCAG | GCAAAGCCCACTTTATTGAGTGC | CCCAGCTTATCTCCTCCTCC |
| rs1143633 | CATCAGACTTTGACCGTATATGCTCAG | TGATCGTACAGGTGCATCGT | TCCAAGAAATCAAATTTTGCC |

Table S5 The multiplex PCR Reaction System of 9 SNPs

|  | Volume |  |
| --- | --- | --- |
| Primer Mix | 4μL | Sangon Biotech (Shanghai) |
| Mg2+ | 1.6μL |  |
| dNTP Mix | 0.4μL |  |
| ExTaq(HS) | 10μL | TAKARA BIOTECHNOLOGY(DALIAN) |
| Template | 2μL |  |

Reaction conditions of PCR: 95ºC 10s+(95ºC 10s+59 ºC40s+72 ºC 20s) * 40+72 ºC10min+4ºC ∞

Table S6 Snapshot multiplex single base extension reaction system

|  | Volume |  |
| --- | --- | --- |
| Snapshot Multiple Kit (ABI) | 5μL | Applied Biosystems |
| Purified multiplex PCR products | 2μL |  |
| Extension Primer (10 μmol/L) | 1μL | Sangon Biotech (Shanghai) |

Reaction conditions: 95°C 10s + (95°C 10s+50°C 5s+ 60°C 30s) * 35 + 60°C 30s+ 4°C ∞
