# Supplementary material for: IL1B polymorphism is associated with essential tremor in Chinese population
Source: BMC Neurol. 2019 May 15;19:99. doi: 10.1186/s12883-019-1331-5 (PMC6518722; doi:10.1186/s12883-019-1331-5)
Supplement: Supplementary file 2 — linkage disequilibrium (LD) of SNPs in the same chromosome. (DOCX 13 kb) [file 12883_2019_1331_MOESM2_ESM.docx]

Information of Linkage Disequilibrium of *IL1B*

| Locus 1 | Locus 2 | D' | LOD | r^2^ | D' conf. bounds | Distance (bp) |
| --- | --- | --- | --- | --- | --- | --- |
| rs1143643 | rs1143634 | 1 | 5.32 | 0.036 | (0.7, 1) | 2088 |
| rs1143643 | rs1143633 | 0.988 | 105.47 | 0.694 | (0.95, 1) | 2165 |
| rs1143634 | rs1143633 | 0.903 | 4.51 | 0.041 | (0.59, 0.98) | 77 |

Information of Linkage Disequilibrium of *NOS1*

| Locus 1 | Locus 2 | D' | LOD | r^2^ | D' conf. bounds | Distance (bp) |
| --- | --- | --- | --- | --- | --- | --- |
| rs7977109 | rs693534 | 0.63 | 3.97 | 0.042 | (0.39, 0.79) | 54378 |
