# Supplementary material for: IL1B polymorphism is associated with essential tremor in Chinese population
Source: BMC Neurol. 2019 May 15;19:99. doi: 10.1186/s12883-019-1331-5 (PMC6518722; doi:10.1186/s12883-019-1331-5)
Supplement: Supplementary file 3 — Demographic Information of Cases (all ET patients) and Controls (DOCX 12 kb) [file 12883_2019_1331_MOESM3_ESM.docx]

Demographic Information of Cases (all ET patients) and Controls

|  | ET sufferers (N=225) | Non-ET/RLS controls (N=229) | P value |
| --- | --- | --- | --- |
| Gender, female, N (%) | 117 (52.00) | 136 (59.39) | 0.113 |
| Age, mean (SD), years | 65.72 (9.25) | 64.30 (13.30) | 0.188 |
| Familial history, N (%) | 100 (44.44) | - | - |
| RLS, N (%) | 25 (11.11) | - | - |

ET= essential tremor. RLS= restless legs syndrome
