# Supplementary material for: IL1B polymorphism is associated with essential tremor in Chinese population
Source: BMC Neurol. 2019 May 15;19:99. doi: 10.1186/s12883-019-1331-5 (PMC6518722; doi:10.1186/s12883-019-1331-5)
Supplement: Supplementary file 4 — The effect of age and sex, and their interaction with each SNP (ET without RLS) (DOCX 19 kb) [file 12883_2019_1331_MOESM4_ESM.docx]

The effect of age and sex, and their interaction with each SNP (ET without RLS)

| Gene | SNP | Dominant model | Adjusted OR (95% CI) | P value | P interaction for sex | P interaction for age |
| --- | --- | --- | --- | --- | --- | --- |
| *HMOX1* | rs2071746 | TT | 1 [ref] | \ | 0.839 | 0.560 |
|  |  | AA+AT | 0.86 (0.56, 1.32) | 0.503 |  |  |
|  |  | Sex, male | 1.53 (1.04, 2.26) | 0.031 |  |  |
|  |  | Age | 1.01 (1.00, 1.03) | 0.143 |  |  |
| *HMOX2* | rs1051308 | AA | 1 [ref] | \ | 0.574 | 0.996 |
|  |  | GG+GA | 0.93 (0.62, 1.37) | 0.701 |  |  |
|  |  | Sex, male | 1.51 (1.02, 2.22) | 0.038 |  |  |
|  |  | Age | 1.01 (1.00, 1.03) | 0.168 |  |  |
| *VDR* | rs731236 | TT | 1 [ref] | \ | 0.833 | 0.279 |
|  |  | CC+CT | 1.51 (0.82, 2.80) | 0.188 |  |  |
|  |  | Sex, male | 1.57 (1.06, 2.31) | 0.024 |  |  |
|  |  | Age | 1.01 (1.00, 1.03) | 0.141 |  |  |
| *IL17A* | rs8193036 | CC | 1 [ref] | \ | 0.682 | 0.154 |
|  |  | TT+CT | 0.98 (0.66, 1.44) | 0.909 |  |  |
|  |  | Sex, male | 1.57 (1.07, 2.32) | 0.022 |  |  |
|  |  | Age | 1.01 (1.00, 1.03) | 0.168 |  |  |
| *IL1B* | rs1143643 | AA | 1 [ref] | \ | 0.624 | 0.931 |
|  |  | GG+GA | 0.87 (0.57, 1.33) | 0.516 |  |  |
|  |  | Sex, male | 1.55 (1.05, 2.29) | 0.026 |  |  |
|  |  | Age | 1.01 (1.00, 1.03) | 0.213 |  |  |
|  | rs1143634 | CC | 1 [ref] | \ | 0.617 | 0.821 |
|  |  | TT+CT | 1.68 (0.71, 3.96) | 0.234 |  |  |
|  |  | Sex, male | 1.55 (1.05, 2.28) | 0.027 |  |  |
|  |  | Age | 1.01 (1.00, 1.03) | 0.162 |  |  |
|  | rs1143633 | AA | 1 [ref] | \ | 0.738 | 0.972 |
|  |  | GG+GA | 1.01 (0.68, 1.50) | 0.948 |  |  |
|  |  | Sex, male | 1.54 (1.04, 2.27) | 0.029 |  |  |
|  |  | Age | 1.01 (1.00, 1.03) | 0.164 |  |  |
| *NOS1* | rs693534 | GG | 1 [ref] | \ | 0.033 | 0.764 |
|  |  | AA+GA | 0.96 (0.65, 1.41) | 0.829 |  |  |
|  |  | Sex, male | 1.57 (1.07, 2.32) | 0.022 |  |  |
|  |  | Age | 1.01 (1.00, 1.03) | 0.167 |  |  |
|  | rs7977109 | AA | 1 [ref] | \ | 0.934 | 0.682 |
|  |  | GG+GA | 0.98 (0.66, 1.46) | 0.938 |  |  |
|  |  | Sex, male | 1.57 (1.07, 2.32) | 0.023 |  |  |
|  |  | Age | 1.01 (1.00, 1.03) | 0.166 |  |  |
| *ADH1B* | rs1229984 | AA | 1 [ref] | \ | 0.022 | 0.227 |
|  |  | GG+GA | 0.99 (0.67, 1.46) | 0.946 |  |  |
|  |  | Sex, male | 1.42 (0.96, 2.09) | 0.076 |  |  |
|  |  | Age | 1.02 (1.00, 1.03) | 0.067 |  |  |
| Gene | SNP | Recessive model | Adjusted OR (95% CI) | P value | P interaction for sex | P interaction for age |
| *HMOX1* | rs2071746 | TT+AT | 1 [ref] | \ | 0.979 | 0.284 |
|  |  | AA | 0.90 (0.55, 1.47) | 0.671 |  |  |
|  |  | Sex, male | 1.54 (1.05, 2.28) | 0.028 |  |  |
|  |  | Age | 1.01 (1.00, 1.03) | 0.140 |  |  |
| *HMOX2* | rs1051308 | AA+GA | 1 [ref] | \ | 0.047 | 0.415 |
|  |  | GG | 1.09 (0.61, 1.96) | 0.762 |  |  |
|  |  | Sex, male | 1.50 (1.02, 2.21) | 0.039 |  |  |
|  |  | Age | 1.01 (1.00, 1.03) | 0.180 |  |  |
| *IL17A* | rs8193036 | CC+CT | 1 [ref] | \ | 0.948 | 0.700 |
|  |  | TT | 1.22 (0.64, 2.34) | 0.549 |  |  |
|  |  | Sex, male | 1.56 (1.06, 2.31) | 0.024 |  |  |
|  |  | Age | 1.01 (1.00, 1.03) | 0.165 |  |  |
| *IL1B* | rs1143643 | AA+GA | 1 [ref] | \ | 0.618 | 0.522 |
|  |  | GG | 1.86 (1.12, 3.11) | 0.017 |  |  |
|  |  | Sex, male | 1.56 (1.06, 2.31) | 0.026 |  |  |
|  |  | Age | 1.01 (1.00, 1.03) | 0.211 |  |  |
|  | rs1143633 | AA+GA | 1 [ref] | \ | 0.865 | 0.873 |
|  |  | GG | 2.57 (1.38, 4.81) | 0.003 |  |  |
|  |  | Sex, male | 1.56 (1.05, 2.31) | 0.026 |  |  |
|  |  | Age | 1.01 (1.00, 1.03) | 0.156 |  |  |
| *NOS1* | rs693534 | GG+GA | 1 [ref] | \ | 0.626 | 0.388 |
|  |  | AA | 1.33 (0.63, 2.82) | 0.455 |  |  |
|  |  | Sex, male | 1.56 (1.06, 2.30) | 0.025 |  |  |
|  |  | Age | 1.01 (1.00, 1.03) | 0.161 |  |  |
|  | rs7977109 | AA+GA | 1 [ref] | \ | 0.421 | 0.686 |
|  |  | GG | 0.74 (0.29, 1.86) | 0.519 |  |  |
|  |  | Sex, male | 1.57 (1.07, 2.32) | 0.023 |  |  |
|  |  | Age | 1.01 (1.00, 1.03) | 0.157 |  |  |
| *ADH1B* | rs1229984 | AA+GA | 1 [ref] | \ | 0.362 | 0.488 |
|  |  | GG | 1.58 (0.76, 3.25) | 0.219 |  |  |
|  |  | Sex, male | 1.43 (0.97, 2.11) | 0.072 |  |  |
|  |  | Age | 1.01 (1.00, 1.04) | 0.065 |  |  |
