# Supplementary material for: IL1B polymorphism is associated with essential tremor in Chinese population
Source: BMC Neurol. 2019 May 15;19:99. doi: 10.1186/s12883-019-1331-5 (PMC6518722; doi:10.1186/s12883-019-1331-5)
Supplement: Supplementary file 5 — Subgroup analysis of association between rs1143633 and ET risk in recessive model (DOCX 15 kb) [file 12883_2019_1331_MOESM5_ESM.docx]

Subgroup analysis of association between rs1143633 and ET risk in recessive model

|  | Recessive model (*IL1B* rs1143633) | Unadjusted OR (95% CI) | P value | Adjusted OR (95% CI) | P value |
| --- | --- | --- | --- | --- | --- |
| ET without RLS | Total cohort |  |  |  |  |
|  | AA+GA | 1 [ref] | \ | 1 [ref] | \ |
|  | GG | 2.52 (1.35, 4.68) | 0.003 | 2.57 (1.38, 4.81) | 0.003 |
|  | Female |  |  |  |  |
|  | AA+GA | 1 [ref] | \ | 1 [ref] | \ |
|  | GG | 2.45 (1.09, 5.49) | 0.027 | 2.45 (1.09, 5.51) | 0.030 |
|  | Male |  |  | 1 [ref] | \ |
|  | AA+GA | 1 [ref] | \ |  |  |
|  | GG | 2.73 (1.02, 7.32) | 0.039 | 2.74 (1.02, 7.38) | 0.046 |
| ET with concomitant RLS | Total cohort |  |  |  |  |
|  | AA+GA | 1 [ref] | \ | 1 [ref] | \ |
|  | GG | 2.59 (1.42, 4.75) | 0.002 | 2.63(1.43, 4.83) | 0.002 |
|  | Female |  |  |  |  |
|  | AA+GA | 1 [ref] | \ | 1 [ref] | \ |
|  | GG | 2.62 (1.20, 5.71) | 0.013 | 2.61 (1.20, 5.70) | 0.016 |
|  | Male |  |  |  |  |
|  | AA+GA | 1 [ref] | \ | 1 [ref] | \ |
|  | GG | 2.68 (1.01, 7.11) | 0.042 | 2.64 (0.99, 7.02) | 0.052 |
