# Supplementary material for: IL1B polymorphism is associated with essential tremor in Chinese population
Source: BMC Neurol. 2019 May 15;19:99. doi: 10.1186/s12883-019-1331-5 (PMC6518722; doi:10.1186/s12883-019-1331-5)
Supplement: Supplementary file 6 — Comparison of age between ET patients and controls in subgroup analysis (DOCX 13 kb) [file 12883_2019_1331_MOESM6_ESM.docx]

Comparison of age between ET patients and controls in subgroup analysis

|  |  | ET | Controls | Age (*p* value) |
| --- | --- | --- | --- | --- |
| ET without RLS | Total cohort, N | 200 | 229 | 0.156 |
|  | Female , N | 100 | 136 | 0.614 |
|  | Male, N | 100 | 93 | 0.221 |
| ET (included ET with concomitant RLS) | Total cohort, N | 225 | 229 | 0.188 |
|  | Female , N | 117 | 136 | 0.521 |
|  | Male, N | 108 | 93 | 0.232 |
