# Supplementary material for: IL1B polymorphism is associated with essential tremor in Chinese population
Source: BMC Neurol. 2019 May 15;19:99. doi: 10.1186/s12883-019-1331-5 (PMC6518722; doi:10.1186/s12883-019-1331-5)
Supplement: Supplementary file 7 — Association of SNPs of candidate genes and odds ratio to ET risk (all ET patients) (DOCX 17 kb) [file 12883_2019_1331_MOESM7_ESM.docx]

Association of SNPs of candidate genes and odds ratio to ET risk (all ET patients)

| Gene | SNP | HWE *p* value | | MAF (case/control) | | Allele | | | | | |
| --- | --- | --- | --- | --- | --- | --- | --- | --- | --- | --- | --- |
|  |  |  |  |  |  | Minor allele | OR | | 95%CI | | *p* |
| *HMOX1* | rs2071746 | 0.601 | | 0.43/0.46 | | A | 0.88 | | (0.67, 1.14) | | 0.325 |
| *HMOX2* | rs4786504 | 0.047 | | - | | - | - | | - | | - |
|  | rs1051308 | 0.887 | | 0.36/0.37 | | G | 0.97 | | (0.74, 1.27) | | 0.829 |
| *VDR* | rs731236 | 0.196 | | 0.07/0.05 | | C | 1.51 | | (0.85, 2.68) | | 0.157 |
| *IL17A* | rs8193036 | 0.867 | | 0.30/0.30 | | T | 1.03 | | (0.77, 1.37) | | 0.861 |
| *IL1B* | rs1143643 | 0.588 | | 0.46/0.43 | | G | 1.14 | | (0.87, 1.48) | | 0.344 |
|  | rs1143634 | 0.482 | | 0.03/0.02 | | T | 1.57 | | (0.70, 3.53) | | 0.274 |
|  | rs1143633 | 0.200 | | 0.39/0.34 | | G | 1.25 | | (0.95, 1.64) | | 0.112 |
| *NOS1* | rs693534 | 0.978 | | 0.26/0.27 | | A | 0.99 | | (0.73, 1.33) | | 0.927 |
|  | rs7977109 | 0.808 | | 0.22/0.23 | | G | 0.92 | | (0.67, 1.26) | | 0.607 |
| *ADH1B* | rs6413413 | *-* | | *-* | | - | - | | - | | - |
|  | rs1229984 | 0.856 | | 0.32/0.32 | | G | 1.03 | | (0.78, 1.37) | | 0.819 |
| Gene | SNP | Dominant Model (adjusted) | | | | Recessive Model (adjusted) | | | | | Genetic Power |
|  |  | OR | 95%CI | | *p* | OR | | 95%CI | | *p* |  |
| *HMOX1* | rs2071746 | 0.82 | (0.54, 1.24) | | 0.343 | 0.90 | | (0.56, 1.44) | | 0.650 | 0.096 |
| *HMOX2* | rs4786504 | - | - | | - | - | | - | | - | - |
|  | rs1051308 | 0.91 | (0.62, 1.33) | | 0.621 | 0.99 | | (0.56, 1.77) | | 0.981 | 0.052 |
| *VDR* | rs731236 | 1.54 | (0.85, 2.80) | | 0.153 | - | | - | | - | 0.399 |
| *IL17A* | rs8193036 | 0.99 | (0.68, 1.45) | | 0.969 | 1.14 | | (0.60, 2.17) | | 0.681 | 0.051 |
| *IL1B* | rs1143643 | 0.89 | (0.59, 1.35) | | 0.594 | 1.84 | | (1.12, 3.03) | | 0.016 | 0.103 |
|  | rs1143634 | 1.58 | (0.68, 3.65) | | 0.289 | - | | - | | - | 0.609 |
|  | rs1143633 | 1.04 | (0.71, 1.52) | | 0.848 | 2.63 | | (1.43, 4.83) | | 0.002 | 0.122 |
| *NOS1* | rs693534 | 0.93 | (0.64, 1.35) | | 0.687 | 1.18 | | (0.56, 2.50) | | 0.663 | 0.050 |
|  | rs7977109 | 0.94 | (0.64, 1.37) | | 0.731 | 0.75 | | (0.31, 1.83) | | 0.530 | 0.072 |
| *ADH1B* | rs6413413 | *-* | *-* | | *-* | - | | - | | - | - |
|  | rs1229984 | 0.93 | (0.64, 1.36) | | 0.705 | 1.55 | | (0.76, 3.14) | | 0.227 | 0.052 |
