# Supplementary material for: IL1B polymorphism is associated with essential tremor in Chinese population
Source: BMC Neurol. 2019 May 15;19:99. doi: 10.1186/s12883-019-1331-5 (PMC6518722; doi:10.1186/s12883-019-1331-5)
Supplement: Supplementary file 8 — Haplotype association study of IL1B and NOS1 (DOCX 13 kb) [file 12883_2019_1331_MOESM8_ESM.docx]

Haplotype association study of *IL1B*

| rs1143643 | rs1143634 |  |  |
| --- | --- | --- | --- |
| Haplotype | Freq. | Case, Control Ratios | P value |
| Haplotype Associations |  |  |  |
| AC | 0.553 | 0.537, 0.568 | 0.3451 |
| GC | 0.419 | 0.429, 0.410 | 0.5609 |
| GT | 0.028 | 0.034, 0.022 | 0.269 |

| rs1143634 | rs1143633 |  |  |
| --- | --- | --- | --- |
| Haplotype | Freq. | Case, Control Ratios | P value |
| Haplotype Associations |  |  |  |
| CA | 0.634 | 0.609, 0.658 | 0.1287 |
| CG | 0.338 | 0.357, 0.320 | 0.2454 |
| TG | 0.026 | 0.034, 0.019 | 0.1791 |

| rs1143643 | rs1143633 |  |  |
| --- | --- | --- | --- |
| Haplotype | Freq. | Case, Control Ratios | P value |
| Haplotype Associations |  |  |  |
| AA | 0.551 | 0.533, 0.568 | 0.2992 |
| GG | 0.362 | 0.386, 0.339 | 0.1468 |
| GA | 0.085 | 0.076, 0.093 | 0.3726 |

| rs1143643 | rs1143634 | | rs1143633 | |
| --- | --- | --- | --- | --- |
| Haplotype | Freq. | Case, Control Ratios | | P value |
| Haplotype Associations |  |  | |  |
| ACA | 0.551 | 0.533, 0.568 | | 0.2994 |
| GCG | 0.337 | 0.354, 0.320 | | 0.2752 |
| GCA | 0.082 | 0.073, 0.090 | | 0.3624 |
| GTG | 0.025 | 0.031, 0.019 | | 0.2457 |

Haplotype association study of *NOS1*

| rs7977109 | rs693534 |  |  |
| --- | --- | --- | --- |
| Haplotype | Freq. | Case, Control Ratios | P value |
| Haplotype Associations |  |  |  |
| AG | 0.530 | 0.532, 0.529 | 0.9313 |
| AA | 0.244 | 0.250, 0.239 | 0.6895 |
| GG | 0.203 | 0.203, 0.203 | 0.9883 |
| GA | 0.022 | 0.015, 0.029 | 0.1563 |
