# Supplementary material for: IL1B polymorphism is associated with essential tremor in Chinese population
Source: BMC Neurol. 2019 May 15;19:99. doi: 10.1186/s12883-019-1331-5 (PMC6518722; doi:10.1186/s12883-019-1331-5)
Supplement: Supplementary file 9 — The effect of age and sex, and their interaction with each SNP (all ET patients) (DOCX 19 kb) [file 12883_2019_1331_MOESM9_ESM.docx]

The effect of age and sex, and their interaction with each SNP (all ET patients)

| Gene | SNP | Dominant model | Adjusted OR (95% CI) | P value | P interaction for sex | P interaction for age |
| --- | --- | --- | --- | --- | --- | --- |
| *HMOX1* | rs2071746 | TT | 1 [ref] | \ | 0.683 | 0.818 |
|  |  | AA+AT | 0.82 (0.54, 1.24) | 0.343 |  |  |
|  |  | Sex, male | 1.43 (0.98, 2.08) | 0.064 |  |  |
|  |  | Age | 1.01 (1.00, 1.03) | 0.154 |  |  |
| *HMOX2* | rs1051308 | AA | 1 [ref] | \ | 0.782 | 0.681 |
|  |  | GG+GA | 0.91 (0.62, 1.33) | 0.621 |  |  |
|  |  | Sex, male | 1.41 (0.97, 2.05) | 0.075 |  |  |
|  |  | Age | 1.01 (1.00, 1.03) | 0.162 |  |  |
| *VDR* | rs731236 | TT | 1 [ref] | \ | 0.972 | 0.287 |
|  |  | CC+CT | 1.54 (0.85, 2.80) | 0.153 |  |  |
|  |  | Sex, male | 1.46 (1.00, 2.12) | 0.051 |  |  |
|  |  | Age | 1.01 (1.00, 1.03) | 0.142 |  |  |
| *IL17A* | rs8193036 | CC | 1 [ref] | \ | 0.901 | 0.148 |
|  |  | TT+CT | 0.99 (0.68, 1.45) | 0.969 |  |  |
|  |  | Sex, male | 1.48 (1.01, 2.15) | 0.043 |  |  |
|  |  | Age | 1.01 (1.00, 1.03) | 0.144 |  |  |
| *IL1B* | rs1143643 | AA | 1 [ref] | \ | 0.819 | 0.685 |
|  |  | GG+GA | 0.89 (0.59, 1.35) | 0.594 |  |  |
|  |  | Sex, male | 1.44 (0.99, 2.10) | 0.057 |  |  |
|  |  | Age | 1.01 (0.99, 1.03) | 0.199 |  |  |
|  | rs1143634 | CC | 1 [ref] | \ | 0.645 | 0.606 |
|  |  | TT+CT | 1.58 (0.68, 3.65) | 0.289 |  |  |
|  |  | Sex, male | 1.44 (0.99, 2.10) | 0.058 |  |  |
|  |  | Age | 1.01 (1.00, 1.03) | 0.165 |  |  |
|  | rs1143633 | AA | 1 [ref] | \ | 0.686 | 0.767 |
|  |  | GG+GA | 1.04 (0.71, 1.52) | 0.848 |  |  |
|  |  | Sex, male | 1.43 (0.98, 2.09) | 0.061 |  |  |
|  |  | Age | 1.01 (1.00, 1.03) | 0.160 |  |  |
| *NOS1* | rs693534 | GG | 1 [ref] | \ | 0.023 | 0.697 |
|  |  | AA+GA | 0.93 (0.64, 1.35) | 0.687 |  |  |
|  |  | Sex, male | 1.48 (1.01, 2.16) | 0.043 |  |  |
|  |  | Age | 1.01 (1.00, 1.03) | 0.144 |  |  |
|  | rs7977109 | AA | 1 [ref] | \ | 0.971 | 0.500 |
|  |  | GG+GA | 0.94 (0.64, 1.37) | 0.731 |  |  |
|  |  | Sex, male | 1.46 (1.00, 2.13) | 0.050 |  |  |
|  |  | Age | 1.01 (1.00, 1.03) | 0.159 |  |  |
| *ADH1B* | rs1229984 | AA | 1 [ref] | \ | 0.017 | 0.349 |
|  |  | GG+GA | 0.93 (0.64, 1.36) | 0.705 |  |  |
|  |  | Sex, male | 1.32 (0.91, 1.93) | 0.147 |  |  |
|  |  | Age | 1.02 (1.00, 1.03) | 0.077 |  |  |
| Gene | SNP | Recessive model | Adjusted OR (95% CI) | P value | P interaction for sex | P interaction for age |
| *HMOX1* | rs2071746 | TT+AT | 1 [ref] | \ | 0.906 | 0.483 |
|  |  | AA | 0.90 (0.56, 1.44) | 0.650 |  |  |
|  |  | Sex, male | 1.44 (0.99, 2.10) | 0.058 |  |  |
|  |  | Age | 1.01 (1.00, 1.03) | 0.140 |  |  |
| *HMOX2* | rs1051308 | AA+GA | 1 [ref] | \ | 0.026 | 0.571 |
|  |  | GG | 0.99 (0.56, 1.77) | 0.981 |  |  |
|  |  | Sex, male | 1.40 (0.96, 2.04) | 0.077 |  |  |
|  |  | Age | 1.01 (1.00, 1.03) | 0.169 |  |  |
| *IL17A* | rs8193036 | CC+CT | 1 [ref] | \ | 0.753 | 0.848 |
|  |  | TT | 1.14 (0.60, 2.17) | 0.681 |  |  |
|  |  | Sex, male | 1.47 (1.01, 2.15) | 0.046 |  |  |
|  |  | Age | 1.01 (1.00, 1.03) | 0.140 |  |  |
| *IL1B* | rs1143643 | AA+GA | 1 [ref] | \ | 0.420 | 0.863 |
|  |  | GG | 1.84 (1.12, 3.03) | 0.016 |  |  |
|  |  | Sex, male | 1.46 (1.00, 2.14) | 0.049 |  |  |
|  |  | Age | 1.01 (0.99, 1.03) | 0.224 |  |  |
|  | rs1143633 | AA+GA | 1 [ref] | \ | 0.975 | 0.739 |
|  |  | GG | 2.63 (1.43, 4.83) | 0.002 |  |  |
|  |  | Sex, male | 1.46 (1.00, 2.14) | 0.051 |  |  |
|  |  | Age | 1.01 (1.00, 1.03) | 0.183 |  |  |
| *NOS1* | rs693534 | GG+GA | 1 [ref] | \ | 0.563 | 0.380 |
|  |  | AA | 1.18 (0.56, 2.50) | 0.663 |  |  |
|  |  | Sex, male | 1.47 (1.01, 2.15) | 0.046 |  |  |
|  |  | Age | 1.01 (1.00, 1.03) | 0.141 |  |  |
|  | rs7977109 | AA+GA | 1 [ref] | \ | 0.542 | 0.968 |
|  |  | GG | 0.75 (0.31, 1.83) | 0.530 |  |  |
|  |  | Sex, male | 1.46 (1.00, 2.13) | 0.049 |  |  |
|  |  | Age | 1.01 (1.00, 1.03) | 0.153 |  |  |
| *ADH1B* | rs1229984 | AA+GA | 1 [ref] | \ | 0.451 | 0.472 |
|  |  | GG | 1.55 (0.76, 3.14) | 0.227 |  |  |
|  |  | Sex, male | 1.33 (0.91, 1.94) | 0.140 |  |  |
|  |  | Age | 1.02 (1.00, 1.03) | 0.076 |  |  |
