# Supplementary material for: IL1B polymorphism is associated with essential tremor in Chinese population
Source: BMC Neurol. 2019 May 15;19:99. doi: 10.1186/s12883-019-1331-5 (PMC6518722; doi:10.1186/s12883-019-1331-5)
Supplement: Supplementary file 10 — Effects of rs1143633 genotype on brain IL1B expression level. (DOCX 28 kb) [file 12883_2019_1331_MOESM10_ESM.docx]

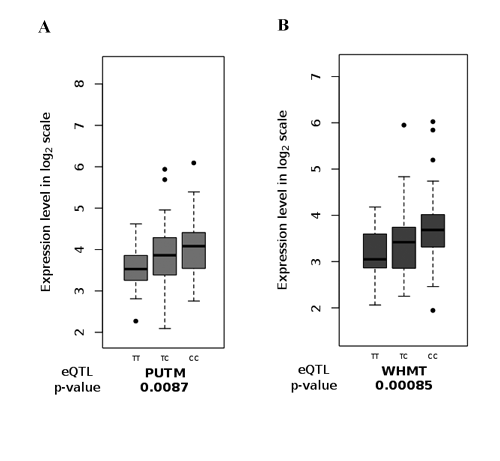


Effects of rs1143633 genotype on brain *IL1B* expression level. (A) The G allele and especially GG genotype increased the *IL1B*expression in putamen with probe set 2571522 (p=8.70$\times$10^-3^). (B) Carriers of G allele and especially GG genotype have more pronounced increased of *IL1B* expression in white matter with probe set 2571524 (p=8.50$\times$10^-4^)
